# Editorial: Preventive and Acute Intervention for Intracranial Atherosclerotic Disease

**DOI:** 10.3389/fneur.2020.00442

**Published:** 2020-06-16

**Authors:** Jang-Hyun Baek, Byung Moon Kim, Simon Chun-Ho Yu, Oh Young Bang

**Affiliations:** ^1^Department of Neurology, School of Medicine, Kangbuk Samsung Hospital, Sungkyunkwan University, Seoul, South Korea; ^2^Department of Radiology, Interventional Neuroradiology, Severance Stroke Center, Severance Hospital, College of Medicine, Yonsei University, Seoul, South Korea; ^3^Department of Imaging and Interventional Radiology, Prince of Wales Hospital, The Chinese University of Hong Kong, Shatin, Hong Kong; ^4^Department of Neurology, School of Medicine, Samsung Medical Center, Sungkyunkwan University, Seoul, South Korea

**Keywords:** intracranial atherosclerosis, endovascular treatment, large vessel occlusion, preventive treatment, ischemic stroke

Intracranial atherosclerotic disease (ICAD) is one of the most important etiologies for acute stroke. Although ICAD has its own distinct characteristics, acute and preventive endovascular treatment (EVT) for ICAD has not been well-studied. Recently, several important ICAD endovascular issues have been developed. First, in the modern era when mechanical thrombectomy has prevailed, ICAD is regarded as a relevant reason for the ineffectiveness of mechanical thrombectomy treatment (1–3). For an acute occlusion by ICAD, different EVT strategies should be elaborated (4). Second, after the SAMMPRIS trial, preventive EVT for ICAD has been discouraged (5). Instead, most patients with ICAD have been treated by intensive medical treatment. To advance preventive EVT for ICAD, it seems to need a breakthrough. This Research Topic discussed the issues for endovascular management of ICAD.

As the first issue, this Research Topic included (1) the method to identify an acute ICAD-related large vessel occlusion (ICAD-LVO) and (2) the EVT strategy for ICAD-LVO. Baek et al. described a unique angiographical way to identify ICAD-LVO before or during EVT—an “occlusion type.” Among the occlusion types, truncal-type occlusion was considered an ICAD-LVO. Truncal-type occlusion was significantly associated with non-embolic occlusion (6, 7). Furthermore, stent retriever thrombectomy was ineffective for the truncal-type occlusion, which needed different types of endovascular modalities other than mechanical thrombectomy devices. In this Research Topic, detailed methodology to assess occlusion type was explained. This review also discussed a few concerns with regard to determining occlusion type. Considering that we do not have reliable information about the etiology of acute LVO before or in the early EVT period, this review might be helpful to set up an optimal EVT strategy. Lee et al. compared occlusion type with the classical definition of ICAD-LVO. The truncal-type occlusion determined by computed tomography angiography was best correlated with fixed focal stenosis in the posterior circulation. This finding is well-understood by the fact that occlusion type in the posterior circulation was less influenced by the degree of pial collateral flow or embolus size, as described in the review by Baek et al. Based on reports by Baek et al. and Lee et al., it seems that those two angiographical surrogate markers for ICAD-LVO should be complementary for a better definition of ICAD-LVO. Besides the angiographical method to identify ICAD-LVO, Bang et al. focused on neuro-imaging features that can aid identification of ICAD-LVO and that should be considered when performing EVT for ICAD-LVO. ICAD-LVO may have different anatomic, hemodynamic, and pathophysiologic features on neuroimaging compared to embolic occlusion. In neuroimaging, ICAD-LVO is associated with intracranial plaques, erythrocyte-poor thrombi, more severe arterial tortuosity with calcification, perforator-bearing segments, and preexisting collaterals, which should be considered when treating ICAD-LVO endovascularly. Even for ICAD-LVO, a stent retriever is still commonly used as the first-line modality. However, we do not exactly know how the stent retriever acts in ICAD-LVO. Lee et al. retrospectively compared outcomes of stent retriever thrombectomy between ICAD-LVO and embolic occlusion. The first-line stent retriever thrombectomy in ICAD-LVO could give a similar immediate successful reperfusion rate to embolic occlusion, although ICAD-LVO finally needed more ICAD-specific modalities such as balloon angioplasty, stenting, or intra-arterial tirofiban infusion. From previous studies, we already know that ICAD-LVO commonly needs the ICAD-specific modalities to achieve a successful recanalization (8–10). This can be also observed in the report by Baek et al.. They compared procedural and clinical outcomes between ICAD-LVO and embolic occlusion in the posterior circulation. Conventional mechanical thrombectomy was less effective in ICAD-LVO. With the use of ICAD-specific modalities, they could get a comparable successful recanalization rate, procedural time, and favorable outcomes for those with embolic occlusions. Such an endovascular strategy based on ICAD-specific modalities was well-summarized in the review by Park et al.. They proposed ways to identify ICAD-LVO, optimal choice of the front-line mechanical thrombectomy modality, and rescue strategy with ICAD-specific modalities after mechanical thrombectomy failure. With all these articles, in ICAD-LVO, successful recanalization could be achieved and time to recanalization could be shorter, which eventually led to better patient clinical outcomes.

As the second issue, this Research Topic included several subtopics on preventive EVT for ICAD. Luo et al. described EVT for ICAD. As we already know, intracranial stenting for secondary prevention of stroke associated with ICAD was not favorable in most clinical situations. However, authors expected that EVT with improved devices and techniques for carefully selected ICAD patients might be beneficial. In fact, Du et al. reported a case series of medically intractable ICAD patients who underwent stenting with Neuroform EZ, a self-expandable stent more easily handled than Wingspan. According to their report, stenting with Neuroform EZ was completely feasible in all patients. Luo et al. also pointed out that perforators around an atherosclerotic lesion is an important factor affecting outcomes of EVT for ICAD. In this Research Topic, Nordmeyer et al. reported that balloon angioplasty with/without stenting was harmful in perforator-bearing atherosclerotic lesions. In particular, periprocedural and follow-up strokes were significantly more frequent in ICAD in the posterior circulation, of which all periprocedural events were perforator strokes.

Differentiation of ICAD from other etiologies (such as arterial dissection, etc.) is also an important issue. Interestingly, Gao et al. reported the case of an acute stroke with an arterial lesion that was evaluated by optical coherence tomography. For their case with basilar artery stenosis, optical coherence tomography gave definite information about arterial dissection and this might be helpful to diagnose ICAD when angiography is ambiguous. In ICAD, vessel wall magnetic resonance imaging can be also helpful. Lee et al. systemically reviewed previous studies of vessel wall magnetic resonance imaging for ICAD. Some imaging findings, such as plaque enhancement, positive remodeling, and plaque surface irregularity were associated with stroke events in patients with ICAD. This finding could be helpful for estimating plaque vulnerability in decision making for ICAD management.

In conclusion, ICAD is obviously a big hurdle in EVT for LVO. For preventive EVT for ICAD, it is a situation that needs successful clinical trials. However, EVT for ICAD will be most likely developed by optimal endovascular strategy and more advanced endovascular devices and techniques. This Research Topic provides an insight into the future prospects of EVT for ICAD.

## Author Contributions

J-HB and BK conceived the manuscript. J-HB drafted the manuscript. BK, SY, and OB critically revised the manuscript. All authors contributed to the article and approved the submitted version.

## Conflict of Interest

The authors declare that the research was conducted in the absence of any commercial or financial relationships that could be construed as a potential conflict of interest.
